# Willingness to pay for informal care in France: the value of funding support interventions for caregivers

**DOI:** 10.1186/s13561-014-0034-2

**Published:** 2014-12-13

**Authors:** Chloé Gervès-Pinquié, Martine M Bellanger, Joel Ankri

**Affiliations:** Management des organisations de santé (MOS), Ecole des Hautes Etudes en Santé Publique (EHESP), Avenue du Professeur Léon-Bernard, 35043 Rennes, France; Laboratoire Santé-Environnement-Vieillissement, Université Versailles-Saint-Quentin, Centre de gérontologie Hôpital Sainte Périne 49 rue Mirabeau, 75016 paris, France

**Keywords:** Support services, Care training, Contingent valuation, Informal care, Alzheimer’s Disease

## Abstract

**Objectives:**

This article aims to assess the relationship between the monetary value of informal care - approximated with the caregiver's willingness to pay to reduce caregiving time - and the caregiver's need of three types of support services: care training, respite care and support group. Developing such services may be the only way to provide sustainable informal care in the future, along with efficient allocation.

**Data & Methods:**

Data used stemmed from two representative national surveys conducted by French National Institute of Statistics and Economic Studies and the French Head Office of Research, Studies, Evaluation and Statistics of the Social Affairs Ministry in 2008. The contingent valuation method was used to approximate the monetary value of informal care. The model was run on 223 informal caregivers of people with Alzheimer's Disease. Statistical analyses were performed using Heckman's two-step estimation strategy, which is known to correct selection bias.

**Results:**

On average, one hour of informal care was estimated at €12.1. Monetary value of informal care was influenced by the caregiver's need of care training (p<0.01). No similar association was found for respite care or support group.

**Discussion:**

Since informal caring value increases with caregivers' need of care training, improving caring skills and capabilities through training support is likely to improve its benefits.

## Background

Total costs of dementia in Europe vary markedly depending on the underlying assumptions that are used in their calculation, for instance in regard to the ‘rather uncertain estimates of informal care costs’ [1]. Costs of dementia were estimated at €160.3 billion in the EU27 in 2008 by Wimo et al. [2] and at €105 billion (€PPP 2010) by Olesen et al. [1]. In Alzheimer’s disease (AD), the most common form of dementia, caregiving costs are primarily accounted for by informal caregivers whose time accounts for most hours of care and assistance [1,3-5].

Because appropriate support is often lacking, caregivers can experience “burden of caring” [6] especially when the need for care provision has not been anticipated [7]. This burden, that can result in impaired caregiver health or opportunity costs, has been defined as a spillover effect by Bobinac et al. [8], as when caring effects are treated as externalities in health economics [9,10]. From a welfare economic perspective, externalities can lead to Pareto inefficiency in AD resource allocation and thus to suboptimal utilization of informal care [11]. Such a situation could be highly detrimental at a time when the population with a strong preference for home care, as in the EU, is ageing and when informal caregiving may become the cornerstone of AD care because of scarce public resources [12,13].

For long-term AD caregiver policies to be effective, the needs of carers for support require better recognition as these needs influence both the carer’s well-being and the resources used in AD care arrangement [14]. For Rosa et al. [14], carers of people with dementia and related disorders experience a large spectrum of needs that include needs for medical and psychological care, social support and education [14]. The policies implemented in most EU countries to support long-term carers are of two main types: financial support measures (aka cash-for-care measures) and in-kind services (e.g. home-based professional services, respite care, counselling, training or support groups) [15-18]. In France, cash-for-care targeting the needs for support of care recipients rather than those of carers prevails [19]; in-kind services are underused [20].

There are effective policies for “alleviating caregiver’s perceived burden and helping prolong the caregiving task” [21], but the evidence for optimal support interventions for carers of people with dementia is scant [22-24]. Moreover, few studies have addressed how carer needs for support affect the economic value of informal care. Because informal care encompasses the intangible value of carer need for support, including both the positive and negative externalities of caring, the estimation method that is used has to be sensitive to carer preferences [25,26]. The contingent valuation method (CVM), which is based on stated preferences, consists in valuing the informal care hour at the ‘price’ the carer would be willing to pay (WTP) to reduce caregiving time by one hour - or would be willing to accept for increasing this time by one hour [26,27]. By finding out carer preferences in relation to caregiving costs, CVM associates carer utility to factors such as their need for support.

The present study highlights funding of care training programmes as a policy driver among support interventions for caregivers in order to improve the benefits of informal care. We asked to what extent an informal carer’s WTP is influenced by their need for in-kind support services (i.e. respite, care training and support groups). Respite can be offered by “taking over informal care temporarily in order to give the caregiver a break and/or enabling the caregiver to participate in other activities” [21]; training covers practical and educational support and information aimed at improving caregiving capabilities; support groups offer collective emotional support.

## Data sources and study sample

### Data sources

Data was collected from The Disability–Health Survey (DHS) and the Disability – Health – Caregiver Survey (DHCS) - two national representative surveys carried out in France in 2008 by the French National Institute of Statistics and Economic Studies (INSEE) and by the French Head Office of Research, Studies, Evaluation and Statistics of the Social Affairs Ministry (DREES). The DHS provides health- and disability-related data for 29,954 people of whom 24,682 are over 18 [28]. The DHCS, the first dataset of its kind in France, provides comprehensive information on the 5,040 informal caregivers of the DHS respondents. A section documents the services specific to informal caregivers (i.e. respite, care training, support groups), caregiver allowances for relief from caregiving (i.e. day care services, professional assistance), and caregiver WTP to be replaced. Each caregiver was assigned an estimation weight based on the generalized weight sharing method enabling estimation of representative results at national level [28].

### Study sample

**Figure 1:**
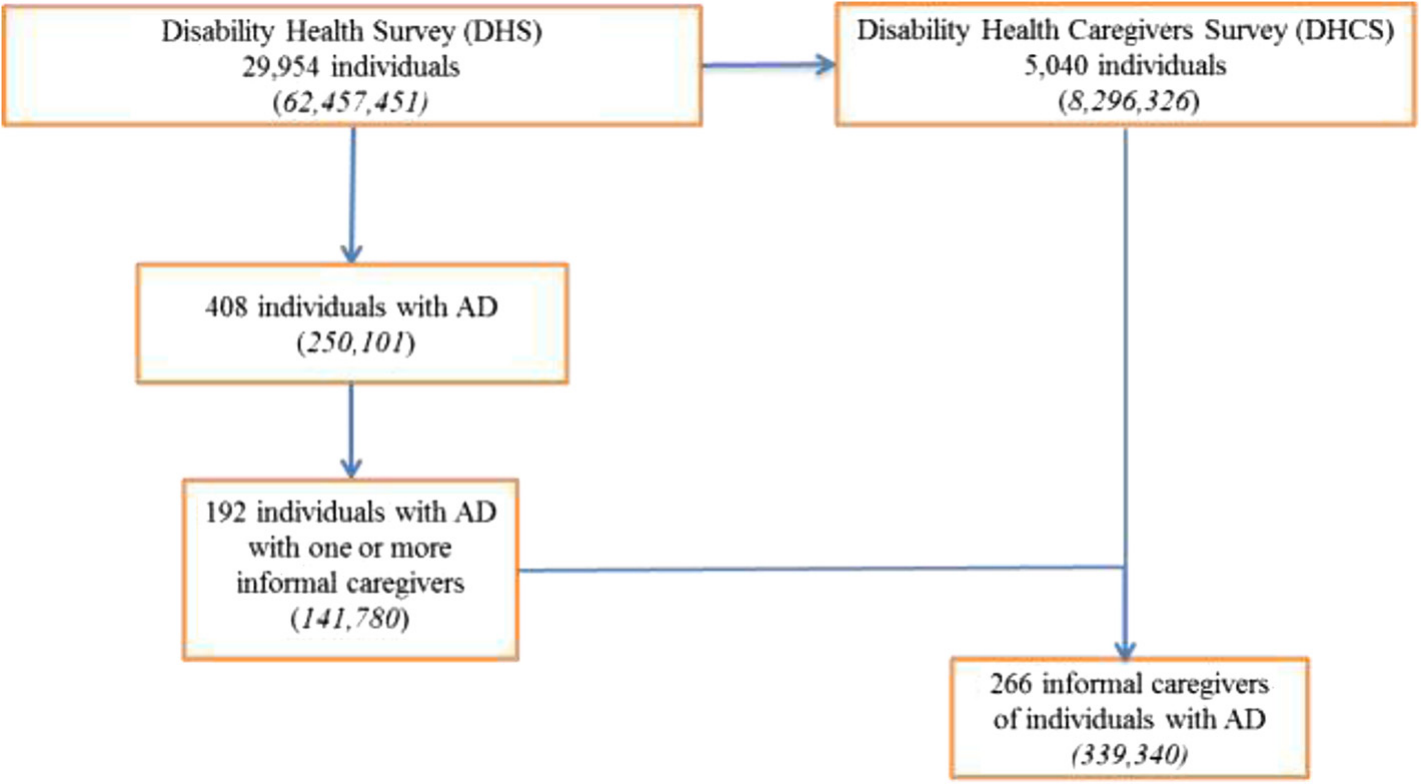
**Selection of informal caregivers caring for individuals with Alzheimer’s disease (AD) (general population figures in parentheses).**

Our study focused on AD caregivers (Figure 1). We selected DHS respondents with memory and orientation disorders, with an alleged diagnosis of AD, who stated that they had at least one informal carer. On merging this DHS sample with the DHCS dataset, we obtained a final sample of 266 informal AD caregivers (72% of AD sufferers had more than one carer). Whether a particular carer had a primary or secondary role in the caregiving process was not known. Given the sample weight, the available information could be assumed to relate to 339,340 informal carers of AD care recipients.

## Methods

### Contingent valuation method and WTP

Caregiver WTP, which expresses the monetary value of informal care and is directly associated with caregiver utility of caring, was estimated using CVM “as a survey-based, hypothetical and direct method for eliciting a monetary value of the support interventions selected” [29]. In the DHCS survey, the question on WTP was an open-ended question:

*“Imagine you could be replaced for one hour for the care you provide to [care recipient’s name]. What is the maximum amount you would be willing to pay for this hour? Before providing an answer, remember that this amount would entail a decrease in your budget”.* In the absence of an answer, a payment card question followed, with seven close-ended ranges of WTP values increasing at fixed intervals.

### Statistical analysis

The impact of the three types of support needs (respite, care training, support groups) on caregiver WTP was analyzed using a Heckman model. Associations between these three needs (dependent variables) and other, dummy variables (carer health, depression, anxiety and sleep disorders) were explored by logistic regression to exclude any significantly associated explanatory variables from the final model and to decrease multicollinearity problems. To deal with multicollinearity, we also ran a proportional odds model for ordinal logistic regression to assess the marginal effects of the relationship between carer and care-recipient health given that carer health was a five-modality categorical variable. To check for potential wealth and caring duration effects on carer WTP or probability to estimate their WTP, t- and chi-square tests were run across both carer income and caring duration groups.

As the response rate to the open question on WTP was only 51%, we checked for protest answers from carers who could not or who refused to give a WTP. After considering a two-hurdle model and a Tobit model in which zero combines “protest zero” and “valid zero”, we chose a Heckman selection model to best address the selection bias of our sample [30]. Because the WTP distribution was asymmetric with a positive skew, we used a log transformation variable Log (WTP +1) to characterize the normal distribution and deal with a zero WTP value in the model.

The Heckman model consisted of two equations. The first (1) was a probit type equation that predicts whether the carer answers the question or not (i.e. the probability of reporting the monetary value of their WTP to be replaced (PRWTP) as estimated by the maximum likelihood method). The second equation (2) was a linear regression equation conditional on the carer providing a response, given by Log (WTP + 1) – LWTP - and regressed by Ordinary Least Squares. The model, when the error terms μ and e are assumed to be bivariate normal with a correlation coefficient ρ = cov[μ,e] ≠ 0, corrects for bias in the estimation of the outcome equation by using the Inverse Mills Ratio (IMR) obtained in the selection model as an explanatory variable. This corresponds to estimated expected error. In other words, sample selection bias was corrected by the selection equation, taking the impact of each observation on the non-random sample into account. After removing observations with missing values, we ran our model for 223 informal caregivers.

Heckman’s 2-stage procedure was specified as follows:

Selection equation for PRWTP:

1 $$ PRWT{P}^{*}=\alpha z+\mu $$

Where *α* is the parameter vector of explanatory variables, PRWTP* is the latent variable of underlying probability of reporting WTP related to the reported binary dependent variable *PRWTP* by the following rule:

$$ PRWTP=\left\{\begin{array}{c}\hfill 1\  if\  PRWT{P}^{*}\ge 0\hfill \\ {}\hfill 0\  if\  PRWT{P}^{*}<0\hfill \end{array}\right. $$

Outcome equation for LWTP:

2 $$ LWTP=\left\{\begin{array}{c}\hfill \beta x+e\  if\  PRWTP=1\ \hfill \\ {}\hfill unobserved\  if\  PRWTP=0\hfill \end{array}\right. $$

*where* β (the parameter vector of explanatory variables) differs from *α* because of exclusion restriction variables included in *α* but not in β. This process has been shown to strengthen identification in the model [31,32]. The key explanatory variables of our model were the three types of carer needs (i.e. respite, care training, support groups). Selected exclusion restrictions were “to benefit from other informal support”, “to already benefit from professional support” and “to lack time for self because of caregiving activities” and were appropriate variables to capture caregiver probability of reporting their own WTP. The fact that the null hypothesis is rejected in performing the Likelihood Ratio test means that the ‘exclusion restrictions’ introduced into our model are relevant, and therefore that our model is relevant.

All statistical analyses were performed using STATA SE-64 statistical software 12.0 (StataCorp. LP, College Station, TX).

## Results

Descriptive statistics for the study’s main variables are given in Table 1. Caregivers were on average 62 (±14.7) years old, predominantly female (62%) (probably spouses or daughters), and tended to be in good health (55%). They cared for an elderly population (mean age: 80 (±8.7) years) in poor health (60.5%) and spent on average 4.1 hours (±4.6) per day on Activities of Daily Living (ADL) and Instrumental Activities of Daily Living (IADL). Half (47.7%) reported to suffer from stress and anxiety and 18-26% said that they were in need of support services. Half had a monthly income of less than €2,000.The estimated mean WTP to reduce caregiving by one hour was €12.1 (±8).

**Table 1:** **Descriptive statistics** (**N** = **266**)

**Informal carer health and socioeconomic characteristics**	
Mean age in years (SD)	62 (14.7)
Male (%)	38.3
Health status (%)	
Very good and good	55
Quite poor, poor and very poor	45
Education level (%)	
Low^a^	50
Middle^b^	37
High^c^	12
Monthly income (%)	
< € 2000	50.44
≥ € 2000	49.56
In work (%)	38.6
**Relationship to care recipient**	
Living together (%)	56.8
Family relationship (%)	
Child	44.4
Spouse	31.2
Other	24.4
Quality of relationship^d^ (%)	
Very good and good	65
Quite poor, poor and very poor	35
**Caregiving activities**	
Help in day/night supervision (%)	68.2/51.9
Managing contacts with formal care (%)	64.6
Help in ADL/ IADL (%)	45.5/46.6
Mean hours of informal care per day (SD)	4.1 (4.6)
Mean years dedicated to informal care (SD)	6.4 (7.1)
**Impact of caregiving on carer** ^**e**^	
Depression (%)	20.6
Sleep problems (%)	32.7
Stress – anxiety (%)	47.7
Neglecting private life (%)	32.7
**Carer self**-**reported need for support services**	
Need for respite care (%)	26
Need for care training (%)	18
Need for support group (%)	20
**Carer WTP to reduce care by one hour**	
Mean WTP (SD)	12.1 (8)
- mean carer monthly income < €2000 (SD)	11.8 (8.6)
- mean carer monthly income ≥ €2000 (SD)	12.4 (7.9)
**Care recipients**	
Mean age in years (SD)	80 (8.7)
Male (%)	36.8
Health status (%)	
Very good and good	39.5
Quite poor, poor and very poor	60.5
Benefiting from professional help	25.8

*Abbreviations*: *SD* standard deviation, *ADL* Activities of Daily Living, *IADL* Instrumental Activities of Daily Living, *WTP* Willingness to pay. ^a^No diploma nor primary school certificate; ^b^Secondary school diploma (baccalaureate); ^c^University degree; ^d^Quality of relationship: 5 categories recoded into 2 categories; ^e^measured using binary variables.

**Figure 2:**
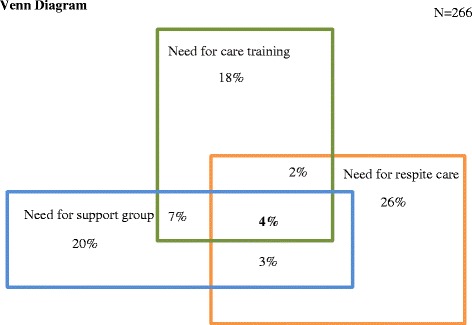
**Venn diagram: overlap between caregivers’ needs for support intervention.**

According to logistic regression analyses, each type of need for support services was significantly and positively associated with one caregiver health-related issue such as sleep disorders or depression (Table 2). In a Venn diagram (Figure 2), there was only a small overlap between the three different types of support needs, indicating few collinearity problems.

**Table 2:** **Impact of caregiver health status on need for support services in multiple bivariate models**

**Carer health status**	**Need for respite care**			**Need for care training**			**Need for support group**		
	**OR**	**SE**	**SD**	**OR**	**SE**	**SD**	**OR**	**SE**	**SD**
General health (5 categories^a^)	1.74**	.320565	-.44013 1.0428	.681	.1516	-.81944 .05254	.87	.18052	-.53753 .2709
Depression (yes vs no)	1.35	.511376	.19798 .91741	1.96	.8309	-.1573 1.5039	2.44*	.97593	.11381 1.6766
Sleep disorders (yes vs no)	1.27	.439664	-.26351 1.1123	2.22*	.8640	-.44018 1.1765	1.97	.73080	-.36302 1.1905
Anxiety (yes vs no)	1.52	.53654	-.43124 .91913	1.44	.5960	.03581 1.5605	1.51	.59943	-.04514 1.4057

*Abbreviations*: *OR* Odds ratio, *SE* standard error, *SD* standard deviation; *p < 0.05; **p < 0.01.

^a^Highest value represents poorest health.

Because care recipient and caregiver health were positively associated (p < 0.05) in an ordered logistic regression, only the explanatory variable ‘care recipient health’ was included in the Heckman model. Because there was no significant difference in caregiver mean WTP value and the probability of reporting WTP between caregivers with an income above or below € 2000, or between caregivers having spent more or less than four years in caregiving, income level or years spent caring were unlikely to distort the results of the Heckman two-step procedure significantly (Table 3).

**Table 3:** **Estimates from a Heckman selection model of informal caregivers**’ **willingness to pay for a reduction in caregiving hours**

	**Selection equation -** **dependent variable:** **PRWTP**			**Outcome equation -** **dependent variable:** **LWTP**		
**Independant variables**	**Coef.**	**Std. Err.**	**Marginal effects**	**Coef.**	**Std. Err.**	**Marginal effects**
Need for respite care (yes vs no)	-.4346946	.2650968		-.3078191	.241379	
Need for care training (yes vs no)	.1123579	.3072039		.5794092*	.2401086	.5199144
Need for support group (yes vs no)	.6392571*	.2831547	.2044924	.249197	.2798483	
Time spent on caregiving (years)	.0043595	.0131211		.0002093	.0107877	
CR^a^ age	-.0125664	.0133103		.0188605	.0142801	
ICr^b^ age	-.0059938	.0102714		-.036121**	.0114112	-.032947
CR sex (male vs female)	.2834009	.2313533		-.0461488	.2146158	
ICr sex (male vs female)	.3725321	.2342962		.2686808	.2316237	
CR health state ( good vs poor)	.1482555	.2161994		-.348649	.2034045	
Help in supervision						
Day supervision (yes vs no)	.1220059	.2909061		-.4346454	.2684354	
Night supervision (yes vs no)	-.2626884	.3038744		.3906264	.274308	
Contact with formal care(yes vs no)	.8182681**	.2340818	.2617563	.3910642	.2708274	
Help in the IADL (yes vs no)	-.3231726	.2284172		.0517261	.2093113	
Help in the ADL (yes vs no)	.1015968	.2264547		-.1422265	.2195051	
ICr monthly income in €	.464248*	.2084316	.1485086	.0984825	.2038006	
ICr relation to CR (partner vs others)	-.1443463	.358649		.9927914**	.3808569	1.151104
ICr and CR live together (yes vs no)	-.3414043	.2921352		-.2461357	.2681222	
Because of care ICr neglects his private life (yes vs no)	.5759702*	.2240331	.1842475	.4168215*	.2163943	
ICr has good relationship with CR (yes vs no)	-.1568137	.0936673		.1926286*	.092616	.2756634
Exclusion restrictions						
CR benefits of professional help (yes vs no)	.5399938*	.2354925	.172739			
CR benefits of another informal help (yes vs no)	-.0936673**	.2257446	-.189248			
ICr doesn’t have enough time for the himself because of care (yes vs no)	-.6227598**	.2465813	-.199215			
Inverse Mills Ratio	.9117173*	.4282136				

PRWTP: Caregivers probability of reporting WTP.

LWTP: Caregivers’ Log (WTP + 1).

^a^Care Recipient.

^b^Informal Caregiver.

*p <0.05; **p <0.01.

No multicollinearity was observed in the model (Variance Inflation Factors were below 2), confirming the results of the Venn diagram. The WTP was positively influenced by need for care training (p < 0.001) but not by need for respite care or for a support group. However, the need for a support group positively affected the probability of reporting WTP (p < 0.05). All three exclusion restrictions, ‘benefiting from other informal support’, ‘already benefiting from professional support’ and ‘lacking time for self because of caregiving activities’ had a significant impact on caregiver probability of reporting their own WTP.

The positive Inverse Mills Ratio (p < 0.05) suggested positive data selection, i.e. carers who estimated their WTP gave a higher WTP than a random population of caregivers with comparable characteristics would have done.

## Discussion

In this first exploration of the influence of informal caregiver needs for support services on the monetary value of informal care, as given by their WTP to reduce their caregiving time, the need for care training was found to increase the monetary value of informal care unlike the need for respite care or participation in support groups.

The divergence in needs may arise from the informal caregiver’s perception of their role. The utility they derive from caring depends on cultural and social dimensions, and this may influence the monetary value they place on their caregiving activity, their choice of being an informal carer, and their use of services. The need for care training probably arises from a keen sense of responsibility, and from a desire for skill development, coping strategies for use in burdensome situations, and competencies for reducing perceived strain. Caregivers with a ‘higher level of mastery of care perceive themselves as able to better answer care demand’ [33-35]. Quality of life, in particular sleep time which was associated with need for care training in our study, is improved [36]. The association between ‘WTP to reduce caregiving’ and ‘care training programmes’ suggests that care training as a support intervention might empower caregivers as reported in the literature on care recipients with cognitive and physical impairment [37-39]. On the other hand, a need for respite or support groups might emphasize the burden of caring including feelings of being overwhelmed or wanting to give up. Provision of respite does not necessarily imply a feeling of respite [40]. Informal carers who are overprotective or take time off may, for instance, feel guilty [41]. In addition, unlike training programmes which not only improve quality of care but also the carer’s process utility, respite care, which just replaces informal care by professional care, or support groups, in which no active individual help is offered to carers, have no direct impact on the caring process [42].

Our informal carers spent on average 4 hours per day (±4.6) on ADL and IADL, supervision (which was associated with PRWTP, p < 0.05), and on managing contacts with formal care. This is in line with the more than 20 hours of care per week reported by Parker for ‘heavily involved caregivers’ [43]. Carer perceived utility and well-being are related to duration and intensity of caregiving, and may thus depend on interventions for improving the overall care process. Care training might help caregivers achieve some “reward” or positive utility and might even improve recipient well-being as coping skills are improved within an altruistic carer-recipient relationship [44-46]. Good carer-recipient relationships (as reported by 65% of our carers) or a positive association between recipient and carer health illustrate this altruistic relationship [44,47].

Our research has economic and policy implications as individual WTPs for informal care were collected in a sample representing 339,340 French caregivers. WTP value is known to be sensitive to total economic value of non-market services, including non-use and intangible values [25,48]. Our estimated monetary value could thus be included in cost-benefit analyses looking for the most efficient AD care arrangement. Our work could lead to replicable studies that include the informal care-related cost and outcomes allowed by WTP elicitation that are usually absent [11]. The marginal effects of need for care training on WTP could inform public decision makers on possible incentives or compensation for improving the benefits of informal care. In line with previous findings [48, 57], our results highlight the value of funding training programmes as drivers for improving the benefits of informal care for both carer and recipient [42,49].

A strong point of our study is that the estimated value of informal care represents the overall value, thus respecting caregiver plurality. Both primary and secondary carers as well as carers who are already supported by professional carer were considered in the DHCS survey and potential interaction effects were controlled via exclusion restriction variables ‘benefits from other informal help’ and “benefits from professional help”. This is important because caring for a single recipient does not necessarily imply a need for the same kind or level of support - especially where two caregivers are siblings, as suggested by Fontaine et al. [50] or because filial norms about care provision differ between daughters and sons of a single care recipient [51]. Another strong point is that heterogeneity in caregiver preferences was taken into account thanks to the sample selection model, as in previous models of preference uncertainty and heterogeneity [27,52].

Our study has several limitations. First, like Koopmanschap et al. [21], we used dichotomous variables to represent support needs. Because intensity of carer preference could not be measured, we could not qualify results in terms of level of caregiver needs. Second, the use of CVM to elicit caregiver preferences in the DHCS is contentious as it supposes that a societal perspective can be adopted by aggregating individual WTPs. Although CVM can be justified by the similarity of WTP elicitation to a market demand schedule, CVM could generate both anchoring effects and opportunistic behaviours [48,53]. Furthermore, because the WTP is estimated for the marginal caregiving hour, it may not be adequate for estimating the total informal care burden. Caution is needed when CVM is the sole method used to advise policy decision makers, with mixed methods using qualitative interviews to capture caregiver motives for refusing to answer the WTP question probably being preferable [54]. Third, provision of support to just a single member of a care recipient - caregiver dyad might not be efficient and the effectiveness of care training thus should be addressed [15,55]. Fourth, the questionnaire did not ask ‘when’ the measure should be implemented, for instance at the onset of caregiving as a burden prevention programme in early-stage AD as suggested by Ryan et al. [56] or only when carers are already experiencing a burden. Lastly, our work focuses exclusively on French caregivers for people with AD living in the community. This might limit its policy implications given the specific context of support service use by caregivers.

## Conclusions

Identifying support interventions for informal caregivers can pave the way for future research that evaluates their cost-effectiveness in a context of a growing number of people with AD and ever scarcer resources. Our findings showed a significant association between the need for care training and the monetary value placed on informal care by caregivers, highlighting caregiver needs to develop their coping skills and competencies and to improve their caring capabilities. Implementing such a policy of care training should lead to a two-fold positive effect, satisfaction of care recipient to be able to stay at home and satisfaction of carers in providing better help to care recipients. Further studies are warranted to ascertain whether these interventions might be both cost-effective and cost-efficient in the long run and relevant to the carer-recipient dyad. Nevertheless, future health economics studies should not overlook needs for respite care and support groups as these are associated with caregiver health. A contingent method to value informal care might not be adequate to reveal their impact on caregiver utility as higher moral constraints might govern these needs than the need for care training.
